# Antiviral Effects of Tecovirimat and Cellular Ultrastructural Changes in Human Bronchial Epithelial Cell Line Following Monkeypox Virus Infection

**DOI:** 10.3390/ijms26062718

**Published:** 2025-03-18

**Authors:** Laura Falasca, Cosmina Mija, Giuseppe Sberna, Massimo Francalancia, Silvia Meschi, Valentina Mazzotta, Enrico Girardi, Andrea Antinori, Fabrizio Maggi, Licia Bordi

**Affiliations:** 1 Laboratory of Electron Microscopy, National Institute for Infectious Diseases Lazzaro Spallanzani—IRCCS, 00149 Rome, Italy; laura.falasca@inmi.it; 2 Laboratory of Virology and Biosafety Laboratories, National Institute for Infectious Diseases Lazzaro Spallanzani—IRCCS, 00149 Rome, Italy; cosmina.mija@inmi.it (C.M.);; 3 Microbiology and Virology Unit, San Gallicano Dermatological Institute—IRCCS, Istituti Fisioterapici Ospitalieri (IFO), 00144 Rome, Italy; 4 Clinical Department, National Institute for Infectious Diseases Lazzaro Spallanzani—IRCCS, 00149 Rome, Italy; valentina.mazzotta@inmi.it (V.M.); andrea.antinori@inmi.it (A.A.); 5 Scientific Direction, National Institute for Infectious Diseases Lazzaro Spallanzani—IRCCS, 00149 Rome, Italy

**Keywords:** mpox disease, MPXV, electron microscopy, mitochondria, tecovirimat, antiviral activity, Calu-3 cells

## Abstract

The mpox virus (MPXV) Clade IIb outbreak in 2022 was the biggest one ever to occur outside Africa, causing different types of clinical symptoms and levels of disease severity. There is no currently approved treatment for mpox, but Tecovirimat has proven effective against known orthopoxviruses in several animal models and Vero cell cultures. Since serious complications, including lung involvement, have been reported, especially in immunocompromised people, we investigated the effects of MPXV infection on the in vitro model of lung airway epithelium (Calu-3 cell line) and examined MPXV replication kinetic and related ultrastructural changes, also performing dose–response studies to measure Tecovirimat antiviral activity. Our results highlighted an active replication of MPXV in Calu-3 cells linked to mitochondrial structural modifications with perinuclear relocation and the formation of cytoplasmic vacuoles. Treatment with Tecovirimat consistently reduced viral replication both in supernatants (81%) and inside cells (77%) and ultimately stopped viral infectivity (92% of cytopathic effect reduction) after 48 h of infection. Drug administration inhibited the final wrapping of mature viral particles, causing extensive cytoplasmic vacuolation. Our results demonstrated Tecovirimat’s in vitro effectiveness against MPXV at the nanomolar concentration on Calu-3 cells. This suggests a potential rationale for using this drug for patients with mpox severe disease and lung involvement.

## 1. Introduction

Mpox (formerly named monkeypox) is a zoonotic disease caused by the mpox virus (MPXV), a double-stranded DNA virus that belongs to the Orthopoxvirus genus in the Poxviridae family [1]. There are two distinct clades of the virus: clade I (with subclades Ia and Ib) and clade II (with subclades IIa and IIb). Before 2022, mpox cases were uncommon outside Africa and mostly related to travel to or from Nigeria [2,3]; since May 2022, mpox cases, mainly caused by clade IIb of the virus, have emerged and spread quickly to about 106 countries where the disease was not endemic, prompting the World Health Organization (WHO) to declare the mpox outbreak a public health emergency of international concern (PHEIC) [4]. Unlike previous outbreaks, this one has involved human-to-human transmission and affected mainly men who have sex with men (MSM) [5,6]. Recently, a new mpox outbreak has emerged, primarily in the Democratic Republic of the Congo and surrounding countries. In response to this outbreak due to subclade Ib of MPXV, the WHO declared mpox again as a global health emergency in August 2024 [7].

After the viral entry into the cell, double-stranded DNA (dsDNA) and viral proteins required for early gene transcription are released into the cytosol. DNA replication then occurs, creating templates for the transcription of intermediate and late genes. The formation of late viral proteins is followed by the assembly of mature intracellular virus (IMV) in the cytoplasm. The envelopment of IMV to form intracellular enveloped virus (IEV) involves various viral proteins, including VP37 protein, which is necessary for forming mature wrapped viruses capable of fusing with the plasma membrane to exit host cells [8].

To date, no specific treatment for mpox exists. However, due to the genetic similarity between the poxvirus causing smallpox and MPXV, the US Food and Drug Administration (FDA) has authorized the emergency use of the anti-smallpox drugs Tecovirimat (ST-246, TPOXX) and Brincidofovir, and the WHO has also added Cidofovir to the list [8,9,10,11]. As for the mechanism of action of these drugs, Cidofovir and Brincidofovir block viral replication, while Tecovirimat, the best-studied among the three antivirals, targets the orthopoxvirus VP37 protein, which is highly conserved and essential for forming and releasing wrapped viruses from infected cells [12]. Its effectiveness against MPXV infection, with good pharmacokinetics, has been demonstrated in animal models [13] and in Vero cell cultures [14]. Although mpox is usually a mild disease with a low case fatality rate, serious complications, such as lung involvement, have been reported with the detection of MPXV DNA in bronchial lavage fluid and tracheal biopsy, especially in immunocompromised patients [15,16]. Ultrastructural studies have described the replication cycle of MPXV in Vero cells and hTERT-immortalized fibroblast cells infected with the strain from the 2022–2023 outbreak and in skin biopsies from infected patients [17,18]. No data of MPXV replication kinetics and ultrastructural changes related to infection on human lung epithelium are still available. Therefore, we examined MPXV replication and ultrastructural changes in Calu-3 cells (a human bronchial adenocarcinoma cell line) and tested Tecovirimat’s antiviral activity to obtain in vitro data that can provide a basis to elucidate pathogenetic mechanisms underlying the pulmonary complications observed especially in immunocompromised subjects.

## 2. Results

### 2.1. MPXV Infection of Calu-3 Cells and Analysis of Cell Ultrastructure

**Figure 1:**
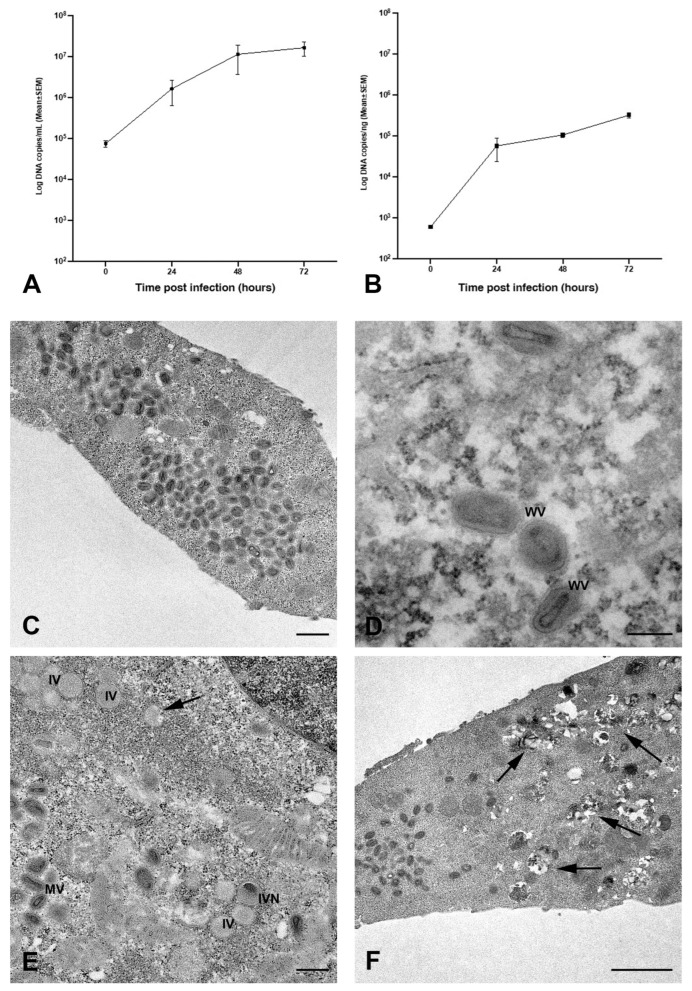
Quantification of MPXV DNA in Calu-3 cultures and transmission electron microscopy images of infected cells. (**A**) Calu-3 cells infected with MPXV at MOI 0.1 and cultured for 72 h at 37 °C. Kinetic of viral yield in cell supernatants quantified by qRT-PCR CPE and expressed as Log copies/mL.(represented as circle) (**B**) Kinetic of intracellular level of MPXV DNA quantified by qRT-PC and expressed as Log copies/ng (represented as square). (**C**–**F**) Representative electron microscopy images of infected cells: (**C**) numerous mature viral particles are visible in the cytoplasm of Calu-3 cells at 48 h.p.i.; (**D**) morphological features of wrapped virions (WV) are shown; (**E**) different steps of MPXV maturation in a cell at 24 h.p.i., crescent (arrow), immature virion (IV), immature particles with eccentric DNA condensation (IVN), and mature virus (MV) displaying typical dumbbell shape of the viral core; (**F**) cytopathic changes induced by MPXV at 72 h.p.i., abundant presence of vesicular structures (arrows) with electron-dense lipidic content, and numerous mature viral particles (MV) are visible. Scale bars: (**C**,**E**) = 500 nm; (**D**,**F**) = 200 nm.

Calu-3 cells, exposed to MPXV at MOI of 0.1, exhibited a gradual rise of viral DNA copies both in the culture medium (Figure 1A) and inside the cells (Figure 1B) throughout the experimental period, indicating that MPXV actively replicates in human bronchial cells.

The transmission electron microscopy (TEM) examination showed the presence of a great number of mature viral particles in the cytoplasm of Calu-3 cells 48 h post-infection (h.p.i.; Figure 1C). MPXV mature particles (MV) were often grouped; most of them displayed an oval shape and contained the typical electron-dense dumbbell core flanked by lateral bodies. Moreover, some wrapped virions (WVs) were observed in the cytoplasm (Figure 1D). The intermediate stages of virus maturation characterizing the cytoplasmic factories were observed (Figure 1E). Groups of immature virions (IVs) appeared as circular structures, enclosed by a membrane containing granular material, some with a displaced nucleoid (IVN). Cytopathic ultrastructural changes caused by MPXV infection were clearly visible at 72 h.p.i., consisting of numerous cytoplasmic vacuoles with membranous or lipidic content (Figure 1F).

**Figure 2:**
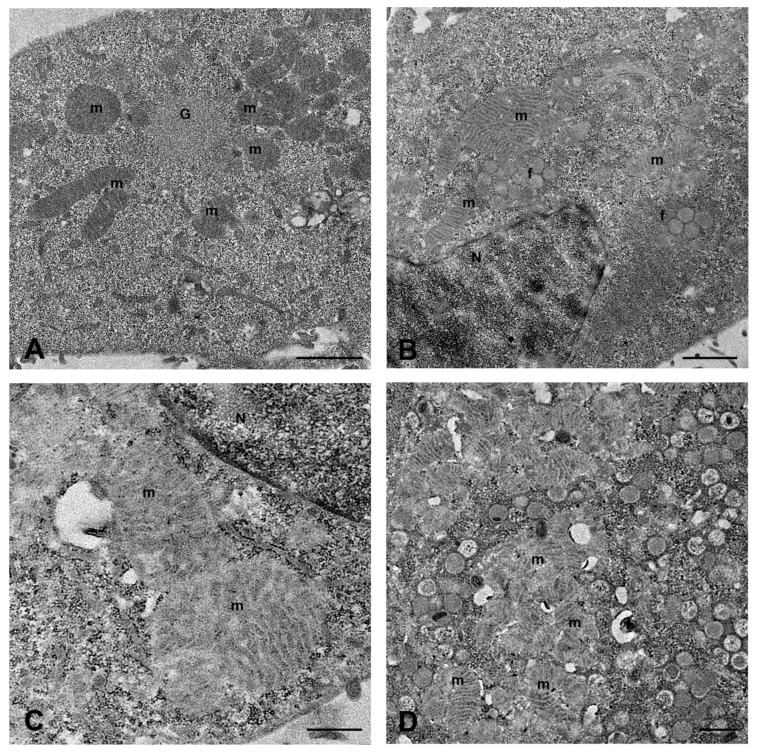
Mitochondria modification in MPXV-infected Calu-3 cells. (**A**) Radial distribution of mitochondria (m) around the granular site (G) of the viral factory. (**B**) Close association of mitochondria with viral factories (f) in the perinuclear area. (**C**) High magnification of large mitochondria near the nucleus. (**D**) Densely packed mitochondria (m), with altered structure, in areas of viral assembly. N = nucleus. Scale bars: (**A**,**B**) = 1 um; (**C**,**D**) = 500 nm.

Significant cell changes were observed concerning mitochondrial distribution and morphology. In the initial stage of viral replication, mitochondria grouped around the granular sites of viral factories, often arranged radially around these regions (Figure 2A). When the assembly of viral particles started, large mitochondria appeared distributed with viral factories in the perinuclear areas (Figure 2B,C). The structure of mitochondria also seemed affected, with the outer membrane becoming faintly visible (Figure 2C), especially at sites of intense viral assembly, where many mitochondria were closely packed and had barely noticeable cristae (Figure 2D).

**Figure 3:**
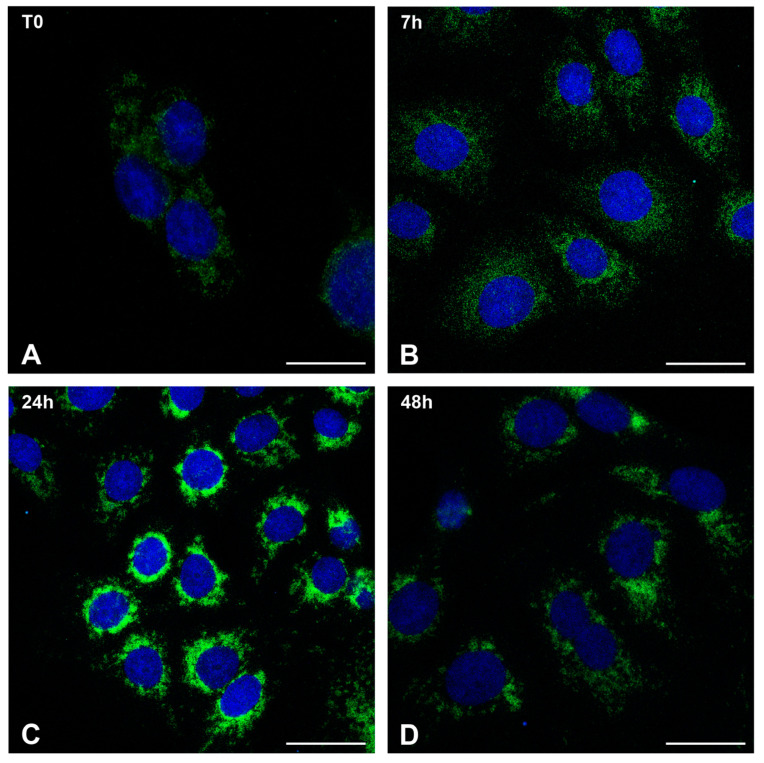
Mitochondria redistribution in MPXV-infected Calu-3 cells. Confocal microscopy representative images of uninfected cells (**A**) and infected cells at different times post-infection (**B**–**D**). Green staining shows localization of mitochondria. Random dots of fluorescence are visible in control cells (**A**) and at 7 h.p.i. (**B**). The mitochondrial network appears strongly modified at 24 h.p.i. (**C**) as highlighted by strong fluorescence localization around cell nuclei (blue). Cells at 48 h.p.i. display more scattered staining (**D**). Staining: Nucleus Blue (DAPI); mitochondria Green (anti-Tim23/Alexafluor488). Scale bars: (**A**–**D**) = 5 μm.

We used confocal microscopy to examine how the mitochondria network changed in infected cells at different time points of infection (Figure 3). Mitochondria were identified using a primary antibody against Tim-23, a mitochondrial localized protein. The biggest change occurred at 24 h.p.i., when mitochondria accumulated in the perinuclear area, as demonstrated by a ring of fluorescence around nuclei (Figure 3C). A more scattered distribution of mitochondria was displayed by some cells at 48 h.p.i. (Figure 3D), when, as demonstrated by an electron microscope, numerous mature virus particles were present.

### 2.2. Tecovirimat Effect on Calu-3 Cells Metabolism

**Figure 4:**
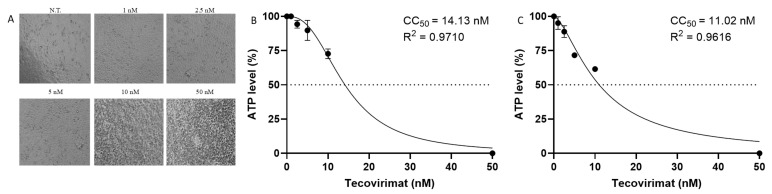
Effect of Tecovirimat on Calu-3 cells. Calu-3 cells were cultured at 37 °C up to 72 h in the absence or presence of scalar doses of Tecovirimat. (**A**) 20× bright-field images Calu-3 cells at 48 h; (**B**) CellTiter-Glo was used to measure the antimetabolic effect of Tecovirimat at 48 h and (**C**) at 72 h.

To test the potential cytotoxicity of Tecovirimat treatment on Calu-3 cells, we cultured the cells up to 72 h at 37 °C in the presence or absence of different drug concentrations (Tecovirimat 1, 2.5, 5, 10, and 50 nM). After 48 h of culture, under a light microscope, we observed a normal adherent phenotype on the surface until the concentration of 50 nM, where a drug-related cytotoxic effect affected the whole monolayer (Figure 4A), being more evident after 72 h. Using Celltiter-Glo to measure the antimetabolic effect of Tecovirimat, we calculated the cytotoxic concentration reducing cell viability by 50% (CC_50_) in Calu-3 cells to be 14.13 nM at 48 h (ranging from 11.71 to 17.14 nM) (Figure 4B) and 11.02 nM at 72 h (ranging from 9.3 to 13.22 nM) (Figure 4C).

### 2.3. Tecovirimat Antiviral Activity on MPXV-Infected Calu-3 Cells

To test the antiviral effect of Tecovirimat, Calu-3 cells were exposed to MPXV with a MOI of 0.1 and grown with or without rising drug levels (1, 2.5, 5, 10, and 50 nM) for up to 72 h at 37 °C.

**Figure 5:**
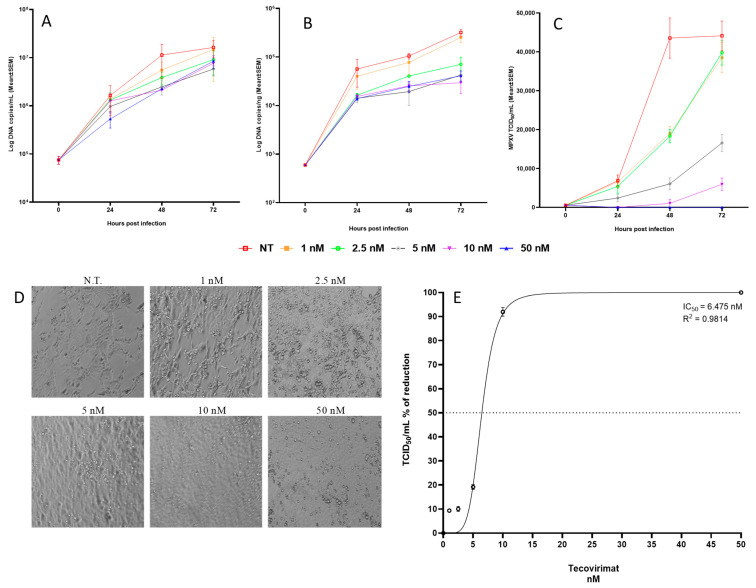
Tecovirimat antiviral activity on Calu-3 cells. Calu-3 cells were infected with MPXV at MOI 0.1 and cultured for 72 h at 37 °C in the absence or presence of scalar doses of Tecovirimat. (**A**) Kinetic of viral yield in cell supernatants quantified by qPCR and results expressed as Log copies/mL. (**B**) Kinetic of MPXV DNA at the intracellular level by qPCR and results expressed as Log copies/ng. (**C**) Viral titer in cell supernatants evaluated as TCID_50_/mL. Experiments were performed as three independent replicates; mean and SEM are shown in the picture. (**D**) 20× bright-field images at 48 h.p.i. (**E**) Half-maximal inhibitory concentration (IC_50_) measured as CPE percentage of reduction at 48 h.p.i.

Treatment with Tecovirimat consistently reduced viral replication in terms of viral genome copies and infectious levels. Specifically, the highest percentage reduction in the viral genome copy number was achieved using 10 nM Tecovirimat both in supernatants (81%; *p* = 0.815, Figure 5A) and at the intracellular level (77%; *p* = 0.078, Figure 5B). Additionally, Tecovirimat exhibited a dose-dependent inhibition of viral infectivity, achieving a significant reduction (92%; *p* = 0.044) at 48 h.p.i. with 10 nM of the drug (Figure 5C,E).

A similar trend was also observed at 72 h.p.i., with a maximal effect obtained at 10 nM of drug (63% reduction in supernatants, *p* = 0.7580; 90.74% reduction at intracellular level, *p* = 0.083; 53.71% of reduction in infectivity, *p* = 0.0177).

Analyzing the results as a whole, the only parameter that showed significant variation both at 48 h and 72 h was the percentage reduction in infectivity when using 10 nM of Tecovirimat, excluding the 50 nM dose due to its toxicity.

Considering the lower toxicity of the drug at 48 h with respect to 72 h and the higher percentage of reduction in infectivity, the best compromise between effective antiviral activity and toxicity seemed to be the use of Tecovirimat 10 nM for 48 h.

The cell shape of cultures observed under a light microscope at 48 h.p.i. showed no protection up to the 2.5 nM dose; partial protection appeared starting from 5 nM, while a normal adherent phenotype on the surface was observed at 10 nM. At 50 nM, the monolayer showed signs of toxicity (Figure 5D). The IC_50_, measured as the percentage of CPE reduction with respect to untreated control virus (considered to be the 100% of infectivity) and calculated by the Reed and Muench method [19], was estimated to be 6.47 nM, ranging from 5.92 to 7.02 nM (Figure 5E).

**Figure 6:**
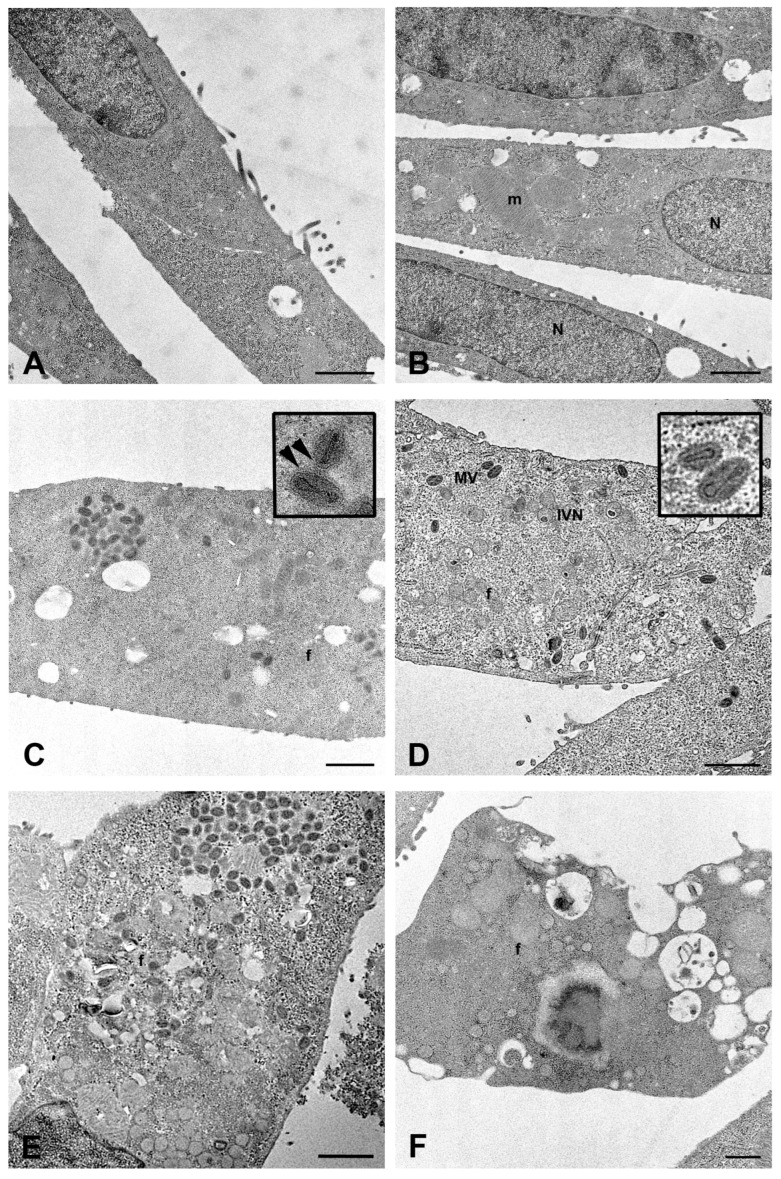
Transmission electron microscopy images of MPXV infection and antiviral treatment with 10 nM. (**A**) Control uninfected and untreated cells after 48 h of culture. (**B**) Uninfected cells cultured in the presence of Tecovirimat for 48 h display normal morphological features. (**C**) Untreated cells 24 h.p.i.; in the insert higher magnification of virions show the presence of envelope (arrowheads). (**D**) Viral factories (f) and few mature virions are visible in Tecovirimat-treated cells after 24 h of MPXV infection; absence of envelope is visible in higher magnification of virions showed in the insert. (**E**) Untreated cells 48 h.p.i. (**F**) Extensive vacuolation in Tecovirimat-treated cells after 48 h of MPXV infection. N = nucleus; m = mitochondria; IVN = immature particles with eccentric DNA; MV = mature virions; f = viral factories. Scale bars: (**A**–**E**) = 1 μm; (**F**) = 500 nm.

The effect of 10 nM Tecovirimat on Calu-3 cells infection was examined at the ultrastructural level (Figure 6). No evidence of drug-induced cytotoxicity was found in the majority of uninfected cells (Figure 6B) compared to uninfected/untreated cells (Figure 6A). After Tecovirimat treatment, few virions were observed in the cytoplasm of MPXV-infected cells at 24 h.p.i., while viral factories were visible (Figure 6D). As expected, compared to enveloped mature viruses observed in untreated cells (Figure 6C), the drug inhibited the final wrapping of mature viral particles (Figure 6D). At 48 h.p.i., MPXV-infected cells treated with Tecovirimat displayed extensive cytoplasmic vacuolation (Figure 6F).

## 3. Discussion

The mpox Clade IIb outbreak in 2022 was the biggest one ever seen outside of Africa, causing different types of symptoms and levels of illness, mainly among MSM and people living with HIV. Since smallpox was wiped out, scientists have kept working on finding antiviral drugs to treat smallpox infections in case of bioterrorism or new related orthopoxviruses that could affect humans [10]. Even though there was no specific treatment for mpox at the time of the 2022 outbreak, Tecovirimat (TPOXX), an antiviral drug approved by the FDA for smallpox, was shown to stop the growth of several orthopoxviruses in laboratory experiments using cells from mice, rabbits, monkeys, and humans and to have a wide range of protective effects in animals that would otherwise die from orthopoxvirus infections [8,20,21,22,23,24,25,26,27]. The ability of Tecovirimat to fight MPXV was also recently tested using Vero E6 and BSC-40 cell lines [12,14,28]. Despite, during the 2022 outbreak, the transmission of MPXV (clade IIb) being mainly through sexual contact [5,6], other routes of transmission have also been described, including airborne transmission, as evidenced by the presence of virus in the air [29,30] and the detection of the viral genome in clinical respiratory samples [2,15,16].

Since severe complications, including lung involvement, have been reported in clinical series [31], especially in immunocompromised persons [15,16,32,33,34], we explored the effect of MPXV infection on a human sub-bronchial gland cell line (Calu-3) and evaluated the in vitro antiviral activity of Tecovirimat. Our results provide further evidence that MPXV productively replicates in Calu-3 cells, confirming data in the literature reporting the susceptibility to the MPXV infection of Calu-3 cells and A549 human lung carcinoma cells as demonstrated by the CPE appearance [35,36] and adding information concerning the kinetic of replication never reported before.

MPXV maturation is a complex process involving various cytoplasmic compartments. In this study, we showed the ultrastructural details of MPXV maturation in a human bronchial epithelial cell line, revealing alterations of mitochondria related to the infection. More in detail, we observed a perinuclear relocation of mitochondria, never described before for MPXV, despite recent publications of electron microscopy analysis in different cell systems [17,28,37,38], raising the question of whether this change could be specific to lung epithelial cells. In this regard, it is worth noting that mitochondria have a key role in airway epithelial function. Lung epithelial cells are often exposed to airborne pathogens, and, being the front line of defense against potential infection, they are the first elements that trigger the signal cascade, which is crucially linked to the onset and development of pulmonary diseases [39]. Mitochondrial changes (organelle physical and functional alterations) in airway epithelial cells were demonstrated to be involved in the pathogenesis of numerous lung diseases [40,41] including viral diseases caused by influenza A virus [42], severe acute respiratory syndrome coronavirus 2 [43], and respiratory syncytial virus (RSV) [44]. A migration of mitochondria to the perinuclear area has been observed during infection with Ectromelia virus (ECTV), the agent of mousepox, which causes severe pneumonia with cytokine storm driven by the TNF signaling pathway [45,46]. Considering this evidence, we could speculate that MPXV might influence the inflammatory response in the lung by altering mitochondria despite more focused investigations being required to understand mitochondrial function better. Nevertheless, this study provided detailed ultrastructural proofs of mitochondrial involvement during MPXV replication in lung epithelial cells.

The results obtained from experiments aimed at establishing the antiviral activity of Tecovirimat showed a dose-dependent inhibition of viral infectivity, achieving a significant percentage of reduction both at 48 h.p.i. (92%; *p* = 0.044) and at 72 h.p.i. (53.71%; *p* = 0.0177) using 10 nM, excluding the 50 nM dose because of its toxicity. Using a 10 nM dose of the drug, a consistent reduction in the number of viral genomes in both supernatants and at the intracellular level was observed at both 48 h.p.i. and 72 h.p.i. However, this reduction was not statistically significant when compared to the untreated control. Notably, the drug does not act directly on viral replication; instead, its action is indirect. The inhibition of the assembly and shedding of new virions results in a reduced number of particles released into the supernatant, which consequently leads to a decrease in the number of infected cells and genome copies at the intracellular level.

Considering the lower toxicity of the drug at 48 h with respect to 72 h and the higher percentage of reduction in infectivity, the best compromise between effective antiviral activity and toxicity seemed to be the use of Tecovirimat 10 nM for 48 h. Therefore, we calculated the IC_50_ at 48 h.p.i., obtaining a value of 6.47 nM, ranging from 5.92 to 7.02 nM.

Our results are in agreement with data from studies performed on BSC-40 cells, indicating a percentage of CPE reduction > 85% using Tecovirimat at 10 nM concentration following infection with 18 clinical isolates of MPXV [47], with IC_50_ ranging from 5.6 to 7.2 nM depending on the strain. Similarly, Warner et al. described the appearance of CPE when using Tecovirimat at doses below 10 nM on Vero E6 cells [10].

For TEM analyses, we used the 10 nM dose at 48 h.p.i. (as we had 92% of reduction in infectivity with an acceptable mortality rate), pushing the system to the limit in order to appreciate ultrastructural changes.

Electron microscopy confirmed that treating Calu-3 cells with 10 nM Tecovirimat reduced mature virus particles within the cytoplasm after 48 h of MPXV infection in line with the drug’s mechanism of inhibiting the virus’s maturation and release. Additionally, MPXV-infected cells exposed to Tecovirimat showed significant cytoplasmic vacuolation; further research is needed to clarify this phenomenon.

## 4. Materials and Methods

### 4.1. Cell and Virus Stock Preparation

Vero E6 (immortalized kidney epithelial cells from the African green monkey) and Calu-3-HTB-55 cells (ATCC) were both kept in Modified Eagle Medium (MEM; Sigma-Aldrich, St. Louis, MO, USA), with 10% Fetal Bovine Serum (FBS; Gibco, Carlsbad, CA, USA) added; all cell lines were grown at 37 °C in a humidified CO_2_ incubator. To prepare virus stocks, Vero E6 cells were infected in the Biosafety Level 3 (BSL3) facility at the National Institute for Infectious Diseases “Lazzaro Spallanzani” (INMI) in Rome with the isolate MPXV virus, human, 2022, Italy, strain hMpxV/Italy/un-INMI-Pt2/2022, clade/lineage IIb B.1 (GISAID: EPI_ISL_13251120, GenBank: ON745215.1), obtained from a skin lesion of an MPXV-infected patient. After being frozen and thawed three times, cell lysates were cleared, aliquoted, and stored at −80 °C. Virus titration was performed on Vero E6 cells with a limiting dilution assay, and the results were shown as a 50% tissue culture infectious dose (TCID_50_/mL). The virus stock titer was 10^7.25^ TCID_50_/mL, equal to 1.2 × 10^7^ Plaque-Forming Units (PFUs). All procedures described below involving infectious MPXV were performed in a BSL3 facility, following standard operating procedures approved by the institutional biosafety committee.

### 4.2. In Vitro Infection Experiments and Tecovirimat Antiviral Activity

Calu-3 cells (passage number 8) were plated at around 250,000 cells/well in 24-well plates, cultured for 24 h at 37 °C and exposed to hMpxV/Italy/un-INMI-Pt2/2022 isolate at a multiplicity of infection (MOI) of 0.1 TCID_50_/cell. After 1 h and 30 min of incubation, the virus was discarded, and cells were rinsed with warm phosphate-buffered saline (PBS) and grown with medium containing 2% FBS with or without gradient doses of Tecovirimat (TargetMol, prodotti Gianni srl, Milano, Italia) (1–2.5–5–10–50 nM) at 37 °C and 5% CO_2_ for up to 72 h. Each treatment was performed in triplicate. At 0 h, 24 h, 48 h, and 72 h cells and supernatants were separately harvested both for infected untreated cells and infected cells treated with gradient doses of Tecovirimat. Viral yield in cells and supernatants was quantified by MPXV DNA PCR, and viral infectivity was assessed in the supernatants, as described below. Experiments were performed in three independent replicates in a BSL3 facility.

### 4.3. Viral Infectivity

Viral infectivity was measured in supernatants of Calu-3-infected cells exposed or not to different doses of Tecovirimat by a limiting dilution assay. More in detail, supernatants collected at various time points of the kinetics were serially diluted in MEM 2% FBS, added to Vero E6 cells in a 96-well plate and incubated at 37 °C for 6 days. The reduction in cytopathic effect (CPE) occurrence was observed by light microscope, and viral titre was calculated according to the Reed and Muench method [19] and expressed as tissue culture infectious dose per ml (TCID_50_/mL).

### 4.4. Detection of MPXV DNA by Real-Time PCR

DNA was extracted from 140 μL of supernatants using the QIAamp Viral DNA Mini Kit (Qiagen, Hilden, Germany) and eluted in 60 μL of Elution buffer. DNA was extracted from cells following the Quik-DNA Microprep Kit protocol (Zymo Research Corporation, Irvine, CA, USA) and eluted in 15 μL of Elution buffer. DNA concentration was measured by NanoDrop™ 2000/2000c Spectrophotometers (Thermo Scientific, Rome, Italy). The amplification was performed using the real-time PCR published by Li et al. [48], targeting the gene encoding the CrmB secreted TNF-alpha-receptor-like protein of the MPXV genome on the m2000 Real-Time System (Abbott, Rome, Italy). A standard, previously quantified by the Bio-Rad QX200 AutoDG Digital Droplet PCR system (Bio-Rad, Hercules, CA, USA) [49], was serially diluted to obtain a curve enabling the transformation of cycle threshold (Ct) values from real-time PCR into quantitative results (qPCR) expressed as copies/mL or copies/ng.

### 4.5. Studies of Tecovirimat Effects on Cell Survival

Calu-3 cells were grown in 96-well plates in MEM with 10% FBS and exposed to different amounts of Tecovirimat (1–2.5–5–10–50 nM) at 37 °C for 48 h. Cell viability was assessed by detecting the ATP levels using CellTiter-Glo (Promega, Madison, WI, USA) to check the toxicity of the drug. Experiments were performed three times.

### 4.6. Dose–Response Curve

The data from the three data sets were averaged to produce dose–response curves. The concentration of Tecovirimat that caused 50% cytotoxicity (CC_50_) and the concentration that inhibited 50% of the effect (IC_50_) were estimated from the curves of concentration versus effect using non-linear regression analysis with GraphPad Prism 9. The IC_50_ of the compound was measured as the percentage of reduction in CPE in supernatants as compared to the untreated control virus (considered to be 100% of infectivity), after 48 h of infection. Results are expressed as TCID_50_/mL and calculated by the Reed and Muench method.

### 4.7. Transmission Electron Microscopy Analysis

Cultured cells were prepared and observed by standard TEM methods. Calu-3 cells were plated at 4 × 10^6^ in T25 flask, cultured for 24 h at 37 °C and exposed to hMpxV/Italy/un-INMI-Pt2/2022 isolate at MOI 0.1; after 1 h and 30 min of adsorption, cells were rinsed with PBS and grown in the absence or presence of Tecovirimat 10 nM. At selected time points (0 h, 7 h, 24 h, 48 h, and 72 h), cells not infected, infected, or infected and treated with Tecovirimat were fixed with 2.5% glutaraldehyde in 0.1 M cacodylate buffer for 4 h at 4 °C. Post-fixation was performed with 1% OsO_4_. Samples were then dehydrated in graded ethanol and embedded in Epon resin. Ultrathin sections were stained with 2% uranyl acetate and observed under a transmission electron microscope JEOL JEM 2100 Plus (Japan Electron Optics Laboratory Co., Ltd., Tokyo, Japan). Images were digitally captured with a digital camera TVIPS (Tietz Video and Image Processing Systems GmbH, Gauting, Germany).

### 4.8. Confocal Microscopy Analysis

For confocal microscopy analysis, cells were seeded in a glass chamber slide (Nunc, Lab-Tek, Waltham, MA, USA) at 120,000 cells/well (1.5 mL/well) and fixed with 4% paraformaldehyde plus glutaraldehyde 0.2% in PBS for 1 h at 4 °C at selected time points (0 h, 7 h, 24 h, and 48 h) after infection with MPXV at MOI 0.1. Samples were washed in PBS, permeabilized with 0.1% Triton X-100 in PBS for 5 min, and blocked with 1% BSA and 10% normal goat serum in PBS for 30 min. The primary antibody, mouse anti-Tim23 antibody (BD Transduction Laboratories, Milano, Italy), a mitochondrial localized protein, was incubated overnight at 4 °C. After washing, samples were incubated with Alexa488-fluorochrome-coupled secondary antibodies directed against mouse (Molecular Probes, Life Technologies, Grand Island, NY, USA). Normal goat serum was used instead of the primary antibody and non-infected samples as appropriate negative controls. Confocal fluorescence microscopy images were obtained using a Zeiss 900 LSM confocal with an Airyscan2 detector and processed using Zen software (version 3.0; Zeiss, Germany).

### 4.9. Statistical Analysis

All figures were graphed using GraphPad 9 (GraphPad Software, La Jolla, CA, USA). Statistical significance between results obtained from untreated cells and treated with different doses of Tecovirimat was calculated by 2-Way ANOVA. *p* values < 0.05 were considered significant.

## 5. Conclusions

Overall, our results demonstrated the in vitro effectiveness of Tecovirimat against MPXV at the nanomolar concentration on human bronchial cell lines. The use of primary cells, Air–Liquid Interface Calu-3 cell culture, or 3D models could represent a crucial step between in vitro studies and clinical trials, allowing ultrastructural changes and drug activity to be verified under conditions that most closely reflect the physiological structure of the lung epithelium.

During the 2022 mpox Clade IIb outbreak, Tecovirimat was primarily used [50], including in pulmonary localization in immunocompromised people [15,16], although there was no definite evidence of effectiveness from clinically controlled studies. Concerning the in vivo viral kinetics during the Tecovirimat exposure of MPXV-infected persons, a progressive decline in viral load was observed throughout the treatment exposure [51]. However, no significant improvement in healing time in Tecovirimat-treated versus untreated was observed, and no difference in time to viral clearance was detected between people assuming Tecovirimat and untreated controls, using counterfactual methods and controlling for confounders [52]. Moreover, the PALM007 trial stated that Tecovirimat was clinically ineffective against MPXV clade 1 in the Democratic Republic of Congo [53]. Further, the STOMP trial, performed in the U.S. and a few other countries with clade II outbreaks and including non-pregnant and non-lactating adults with clade IIb mpox who did not have severe immunocompromise or severe mpox, showed that Tecovirimat was safe but did not reduce the time to resolution of mpox in participants who received Tecovirimat compared to participants who received placebo [54].

Despite these negative results in randomized trials, the role of this drug in the treatment of mpox in patients with severe immunocompromise, such as people with advanced and uncontrolled HIV infection, has not been still determined in controlled studies and requires additional clinical trials focused on people with immunodeficiency.

The results of the current study, confirming the in vitro drug effects against MPXV in lung cell lines, indicate that the controversy over the efficacy of Tecovirimat in severe clinical manifestations, such as lung involvement, is still open. Current data also indicate that while Tecovirimat is generally safe, it requires careful monitoring in this patient population. Clinical trials suggest that further research is necessary to fully understand its effects. As always, a thorough risk–benefit assessment is crucial, given that MPXV infection in severely immunocompromised individuals can be extremely severe, potentially leading to death.

This suggests that evaluating in vitro activity on lung cell lines infected with MPXV clades other than IIb and promoting large randomized controlled trials on immunocompromised persons is urgent to settle whether the drug should be used in an appropriate clinical setting.

## Data Availability

The original contributions presented in the study are included in the article; further inquiries can be directed to the corresponding author.
